# Pin penetration depths in the neurocranium using a three-pin head fixation device

**DOI:** 10.1038/s41598-024-55227-x

**Published:** 2024-02-27

**Authors:** René Machts, Martina Schindler, Heike Unterhauser-Chwastek, Jan Mertens, Katharina Faust

**Affiliations:** 1 Pro Med Instruments GmbH, Part of Black Forest Medical Group, 79111 Freiburg, Germany; 2 Department of Neurosurgery, Bundeswehrkrankenhaus Berlin, 10115 Berlin, Germany; 3 Department of Neurosurgery, Charité University Medicine, 10117 Berlin, Germany

**Keywords:** Bone response, Loss of head fixation, Head fixation device, Head slippage, Skull clamp, Cadaveric specimen, Risk factors, Biomedical engineering

## Abstract

In estimated 10–15% of neurosurgical interventions employing a conventional three-pin head fixation device (HFD) the patient’s head loses position due to slippage. At present no scientifically based stability criterion exists to potentially prevent the intraoperative loss of head position or skull fractures. Here, data on the skull penetration depth both on the single and two-pin side of a three-pin HFD are presented, providing scientific evidence for a stability criterion for the invasive three-pin head fixation. Eight fresh, chemically untreated human cadaveric heads were sequentially pinned 90 times in total in a noncommercially calibrated clamp screw applying a predefined force of 270 N (approximately 60 lbf) throughout. Three head positions were pinned each in standardized manner for the following approaches: prone, middle fossa, pterional. Titanium-aluminum alloy pins were used, varying the pin-cone angle on the single-pin side from 36° to 55° and on the two-pin side from 25° to 36°. The bone-penetration depths were directly measured by a dial gauge on neurocranium. The penetration depths on the single-pin side ranged from 0.00 mm (i.e., no penetration) to 6.17 mm. The penetration depths on the two-pin side ranged from 0.00 mm (no penetration) to 4.48 mm. We measured a significantly higher penetration depth for the anterior pin in comparison to the posterior pin on the two-pin side in prone position. One pin configuration (50°/25°) resulted in a quasi-homogenous pin depth distribution between the single- and the two-pin side. Emanating from the physical principle that pin depths behave proportionate to pin pressure distribution, a quasi-homogenous pin penetration depth may result in higher resilience against external shear forces or torque, thus reducing potential complications such as slippage and depressed skull fractures. The authors propose that the pin configuration of 50°/25° may be superior to the currently used uniform pin-cone angle distribution in common clinical practice (36°/36°). However, future research may identify additional influencing factors to improve head fixation stability.

## Introduction

Most craniotomies and dorsal approaches to the cervical spine are performed employing an invasive head fixation device (HFD) or skull clamp, respectively. The main function of HFDs is to retain the delicate anatomy of the brain and its adjacent structures motionless to the surgeon, while at the same time providing for stable attachments of auxiliary instruments, e.g., retractor systems or neuronavigation accessories.

One common, yet underreported occurrence using HFDs is the intraoperative unintended dislocation of the head, so-called slippage. Slippage may lead to serious complications ranging from neuronavigation inaccuracies, scalp injuries to cranial fractures and epidural hematoma^1–3^. The literature on incidence and extent of cranial slippage is scarce. It is estimated to occur in 10–15% of cases^4,5^. A variety of influencing factors have been put into connection with the likelihood of slippage. Those include forceful maneuvers during bone work, unintentional pressing on the HFD, repositioning of the table intraoperatively and unexpected body movements of the patient^2^. Skull fractures and their resulting comorbidities could be traced back to an inadequate application of pin force^6,7^.

Raabe et al. have identified a safe and expedient pinning zone on the neurocranium based on empirical data and mechanistic considerations^8^. The delineated safe pinning zone, however, has not been corroborated by quantifiable biomechanical data yet.

Potential variables that may alter the mechanical stability of a three-point HFD are the pin-cone angle and the pin-penetration angle in relation to the skull surface, as is mainly implemented through the angulation of the two-pin holding rocker. The prevailing pin configuration in clinical practice (in adult neurosurgery) entails equal pins of 34° to 38° pin-cone angle for all three pin pressure points and an angled two-pin rocker of 40° to 45° assembly.

Considering the following simple physical principles, namely:

1. Force equals counterforce (Newton’s third law),
2. Pressure equals force per area,
3. Penetration depth is proportionate to applied pressure,
4. Non orthogonally applied force exerts less pressure than orthogonally applied force based on Pythagoras law of the vectors,

We hypothesized that:

1. Using the same pin-cone angle both on the single-pin and the two-pin side will result in a deeper bone penetration on the single-pin side (as the counterforce will be roughly distributed between the two opposing pins).
2. Smaller pin-cone angles on the two-pin side opposing one larger pin on the single-pin side may result in a more even pressure distribution.
3. Due to the non-spherical form of the skull with varying local convexity curvatures, different head positions will lead to altering bone penetration depths.

A previous cadaveric study by Visentin et al. analyzed cranial pin penetration depths of two commercially available pin-cone angles (35°: commercial adult pin; 43°: commercial pediatric pin) by means of cone beam CT images, assessing the single-pin side^9^. The group confirmed that the smaller pin-cone angle did result in deeper bone penetrations, and that bone penetration depth generally increased with the applied pin force (180 N to 270 N).

However, biomedical studies aiming at attaining a quasi-homogenous pin pressure distribution to reduce the risk of head dislocation have not been pursued to date. Here, for the first time, we employ various (not commercially available) pin-cone angles between 25° and 55°, as well as asymmetric pin-cone angle distributions between the single-pin and the two-pin side, to potentially identify configurations of higher biomechanical stability. Our study aims at laying a foundation for a stability criterion that may reduce slippage complications in the future.

## Methods

### Ethics and informed consent

The cadaveric specimens were provided through the Institution Charité Berlin “chirurgisch-anatomisches Trainingszentrum” (Centre for Anatomy). The study was approved by the IRB of “Ethikkommission der Charité Universitätsmedizin” (Charité's Ethics Committee) within the Institution Charité Berlin “chirurgisch-anatomisches Trainingszentrum” (Centre for Anatomy). All methods were carried out in accordance with relevant guidelines and regulations. All the deceased utilized here had expressed their wish, as well as given their informed written consent to donating their bodies to the University Clinic for scientific use, including the invasive experiments in the study. An additional vote by the local ethics committee was inapplicable, accordingly.

### Experimental equipment

#### Specimen

Eight fresh, chemically untreated human cadaveric head specimens were used. Specimens were of both sexes (four male, four female) with no further specification of age, ethnicity, or other biological factors. Specimens were stored overnight in a 4 °C temperature and humidity-controlled environment. Specimens with head injuries or prior cranial surgery were excluded from the study. All heads were initially weighted, and their circumferences were documented. Please see Table 1.

**Table 1:** Head weight and head circumference of cadaveric specimens.

Cadaveric specimen	Head weight in g	Head circumference in cm
1	4408	59.0
2	4201	58.0
3	5606	59.5
4	4905	55.5
5	4171	57.0
6	3739	54.0
7	4157	55.5
8	3388	52.0
Mean	4322	56.3
SD	640	2.4

#### Head fixation device (HFD)

**Figure 1:**
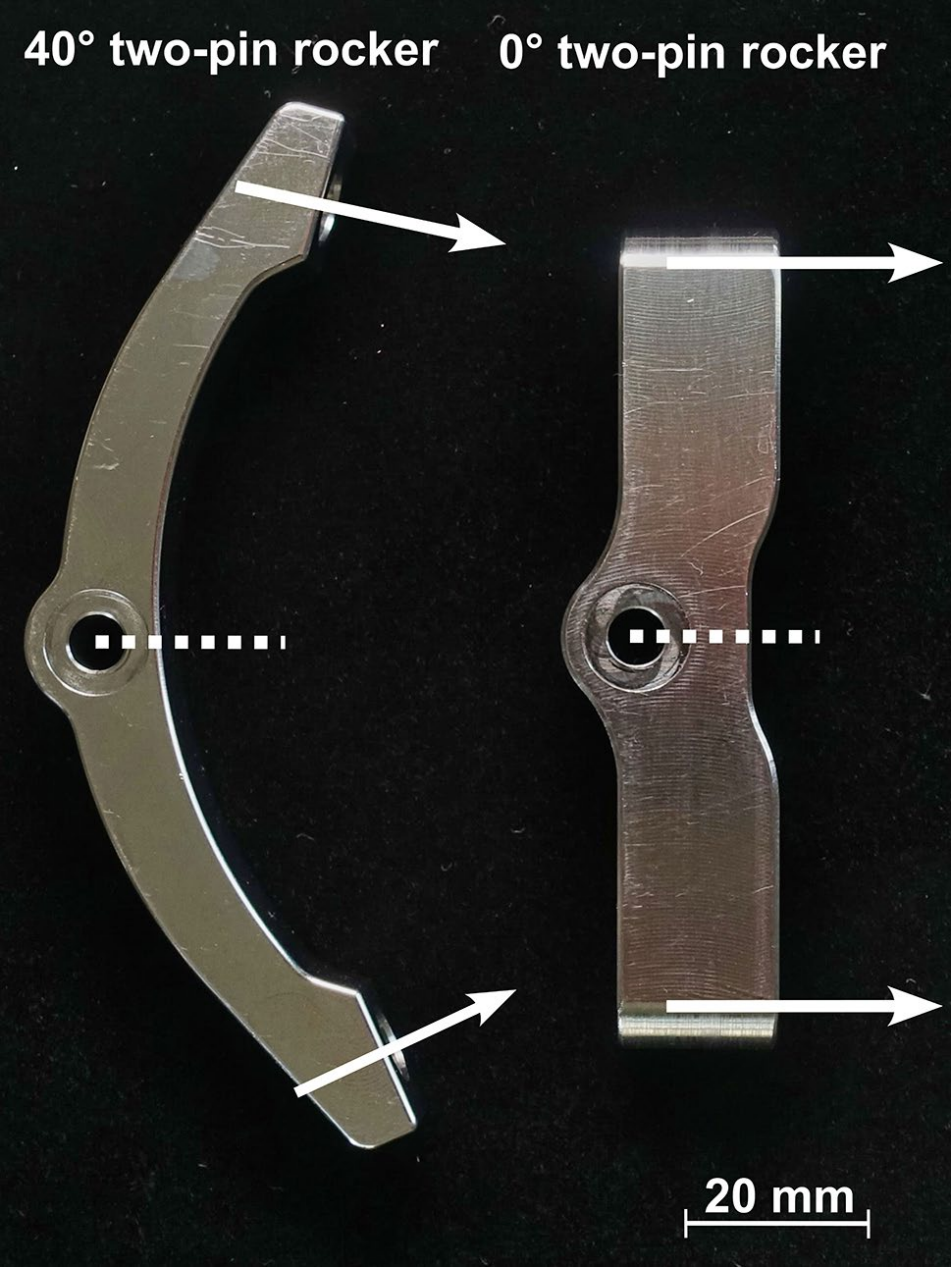
Standard DORO two-pin rocker is shown left. It has a curvature of 40°. Modified two-pin rocker is shown right. It has a curvature of 0°. The distance between the pin tip axes is identical. White arrows symbolize the alignment of skull pins.

A conventional HFD was used for the application of pin force (DORO QR3 skull clamp system, pro med instruments GmbH, Freiburg, Germany). Two two-pin rockers were employed; one of which was a commercially available standard two-pin rocker with a curvature of 40°; the other one was an especially manufactured two-pin rocker with a curvature of 0°. The used two-pin rockers are depicted in Fig. 1.

#### Pins

**Figure 2:**
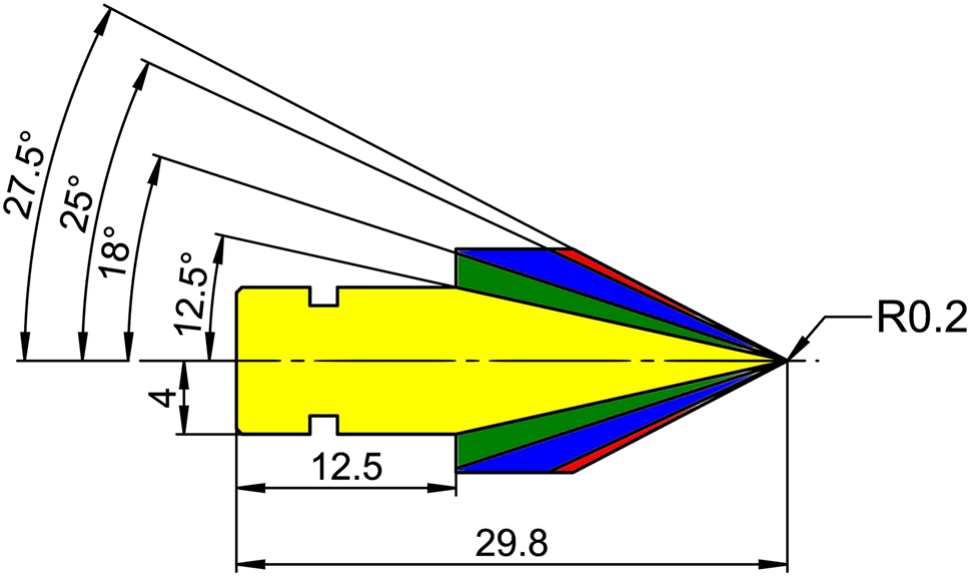
Dimension of pins in mm. The red marked area represents the pin-cone angle of 55°, blue 50°, green 36°, yellow 25°. The pin tip has a radius of 0.2 mm for each pin.

Four skull clamp pins of different pin-cone angles were used interchangeably, each manufactured of the same Ti6Al4V alloy. A commercially available pin was used, which was of the pin-cone angle 36° (DORO skull pin, pro med instruments GmbH, Freiburg, Germany). In addition, the following pin-cone angles were deployed: 25°, 50°, 55°. The geometry of the used pins is depicted in Fig. 2.

### Experimental design

During the test series three variables were alternately exchanged: (1) pin-cone angle, (2) angle of two-pin rocker, (3) head position for craniotomy approach.

1. Pin-cone angle; always depicted as “single-pin side/two-pin side”: five pin configurations were used: 1. 36°/36° (i.e., the routinely used pin configuration of the commercially available adult pins to date); 2. 50°/25°; 3. 50°/36°; 4. 55°/25°; 5. 55°/36°.
2. The two-pin rocker angles (40° the standard and commercially available angle and 0° angle) were combined with each pin configuration and each head position.
3. Three head positions for standard craniotomy approaches were deployed according to Withney et al. ^10^. Those were: 1. prone (cranial bone pinned by single-pin side and two-pin side: parietal bone), 2. middle fossa (cranial bone pinned by single-pin side: frontal bone, and two-pin side: parietal bone), 3. pterional (cranial bone pinned by single-pin side: frontal bone and two-pin side: mastoid process by anterior pin and parietal bone by posterior pin).

**Figure 3:**
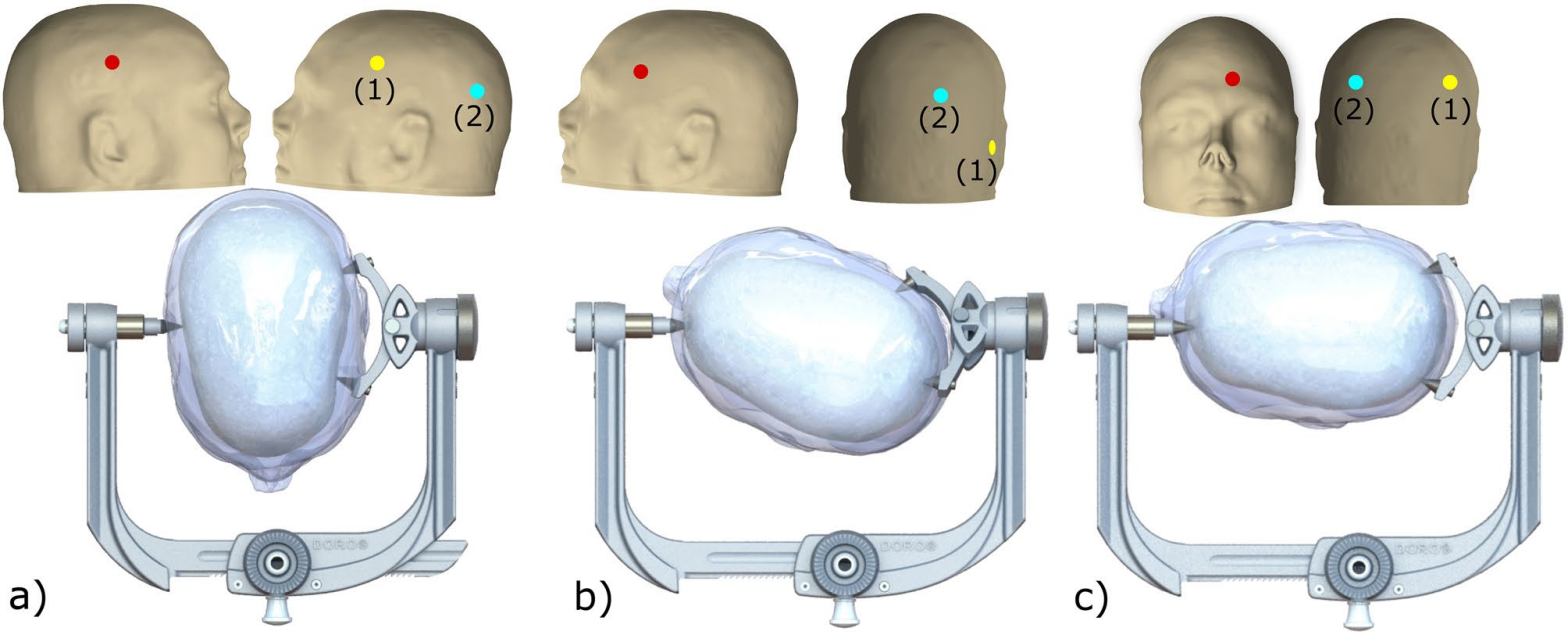
Head positions for craniotomy approach and positions of pins during the application of pin force. Figure (**a**) depicts the head position for prone, (**b**) pterional and (**c**) middle fossa. We randomly changed the single-pin side and two-pin side in our experiment. The red dot represents the position of pin on single-pin side. The yellow dot represents the anterior or right position on two-pin side (1) and the light blue dot the posterior or left position on two-pin side (2). The scalp is transparent in the insets. The adapted human model is a 24 year-old man and originates from Biomedical Image Analysis Group ^11^. We created the figures using 3D Slicer (Version 4.11.20210226, https://www.slicer.org/) as well as MeshLab (Version 2022.02, https://www.meshlab.net/) for creation of head compartment meshes and SOLIDWORKS (Version 2019, https://www.solidworks.com/) for built up the complete assembly and rendering the final figures.

Head angulation and pinning positions on the neurocranium are depicted in Fig. 3. The pins on the two-pin side are discriminated into pin (1) and pin (2). For the respective positional assignment of pin (1) and (2) in each head position please see Fig. 3.

**Figure 4:**
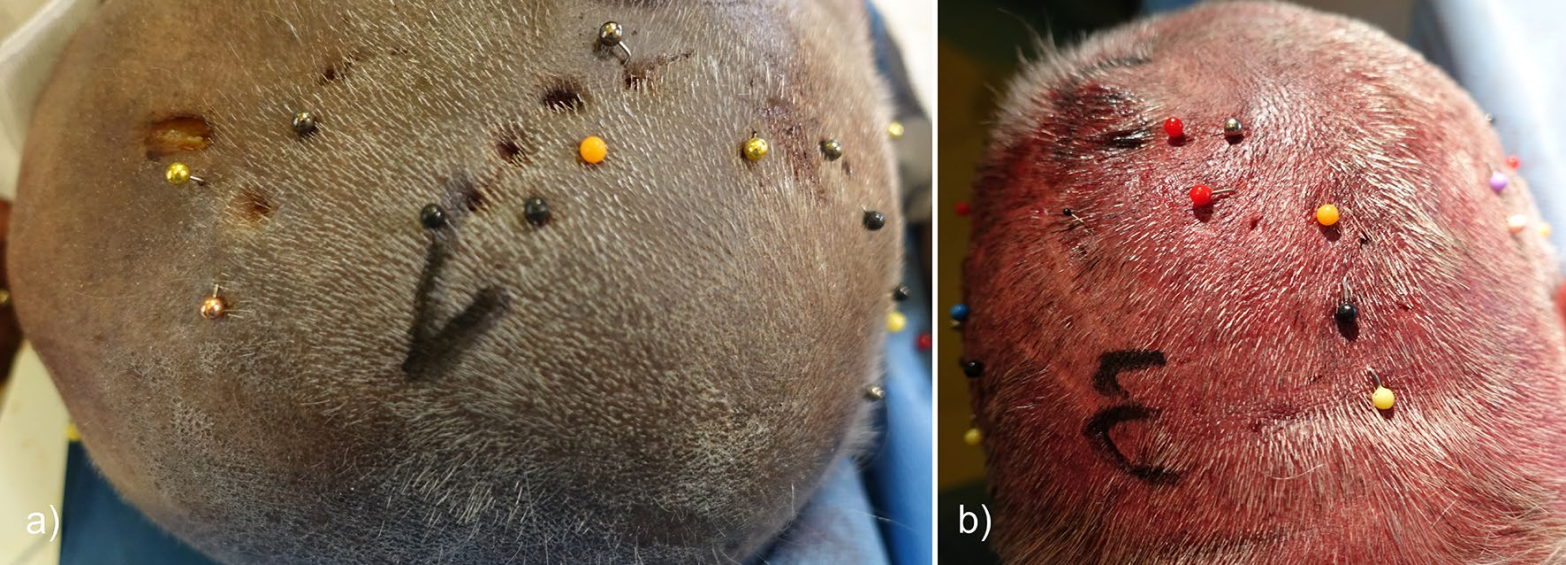
Applied geometric and colored markers on scalp of cadaveric specimen 1 (**a**) and 3 (**b**) in two different views.

Configurations of all three variables resulted in 30 single tests. Each test was repeated three times, producing 90 head pinning procedures in total. A pseudorandomized assignment of test to cadaveric specimens was chosen. For each test, the constant pin force of 269.5 N was applied using a calibrated non-commercial DORO clamping screw. The applied pin force was controlled by a gauge block and not as usual by using the optical force indicator. After application of pin force, geometrical- and color-coded markers were immediately placed near the penetration into the scalp to securely assign the respective penetration to the corresponding test series. Examples of the geometric and colored markers in the scalp of cadaveric specimens are shown in Fig. 4.

### Bone penetration depth measurement

**Figure 5:**
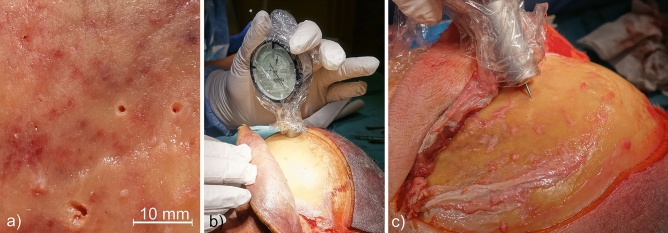
Measurement of penetration depth after removing the scalp. (**a**) The penetration pattern on neurocranium. Three larger punction holes are in the middle and bottom of the picture. One smaller punction hole is in the left corner at the top of the picture. (**b**) The usage of dial indicator. The insertion of dial indicator needle in the penetration depicts (**c**).

After completion of the pinning procedure scalp and periosteum were removed from each cadaveric specimen. This allowed a direct mechanical measurement of penetration depth using a dial indicator (2343-101, INSIZE Co., Ltd, China, accuracy 17 µm) equipped with a measuring stylus smaller than the pin-cone angles. The implemented seating of the dial indicator (R = 8 mm) provided an orthogonal measurement in the neurocranium. An exemplary penetration pattern on neurocranium is shown in Fig. 5a, and the measurement by dial indicator in Fig. 5b,c. Since only orthogonal measurement was technically possible, tangential angles of impact to the bone that resulted in longitudinal scratches had to be omitted from the analysis.

### Statistical analysis

Mean and standard deviation (SD) were reported for continuous variables. The measured penetration depth in dependency of pin-cone angle set up, utilized two-pin rocker and head positions for standard craniotomy approaches were analyzed by the Scheirer–Ray–Hare–test with a Dunn’s Kruskal–Wallis Multiple Comparisons post-hoc test due to the lack of homoscedasticity. The Wilcoxon–Mann–Withney–test was performed to compare the penetration depth of single-pin side with the penetration depth of two-pin side (1) and (2). The significance level was set to ≤ 0.05 to identify significant differences.

## Results

### Main results

There are five relevant findings in this study:

1. The current standard pin configuration in surgical practice (36°/36°) resulted in an asymmetric pin pressure distribution with significantly deeper bone penetration depth on the single-pin side than on the two-pin side.
2. A novel pin configuration was identified that produced quasi-homogenous pin penetration depths. The novel pin configuration is 50° on single-pin side and 25° on each pin on two-pin side.
3. Pin penetration depths varied significantly depending on the head position for standard craniotomy approach.
4. The angle of the two-pin rocker had no influence on bone penetration depth.
5. Skull fractures occurred only on the single-pin side.

### Penetration depth on single-pin side

**Figure 6:**
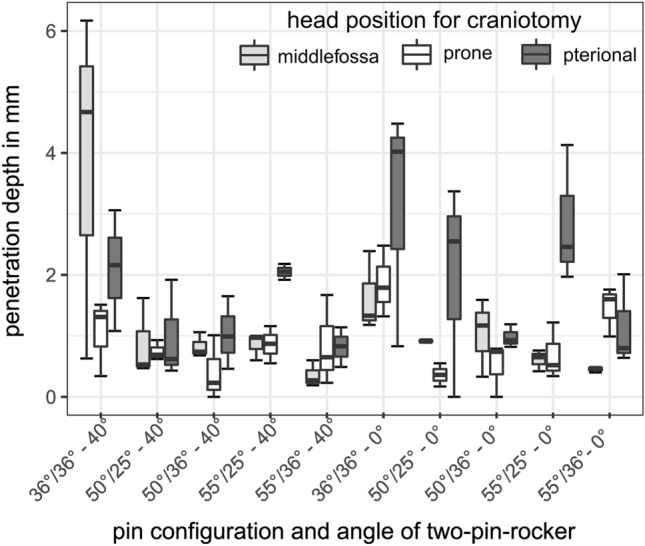
Penetration depth on single-pin side for each test setup. The first number on the bottom describes the pin-cone angle on single-pin side in degree. The second number describes the pin-cone angle on two-pin side in degree. The third number represents the 40° or 0° two-pin rocker.

In three cases bone penetration depth measurement was not possible on the single-pin side based on tangential impacts of the pin to a locally sharp skull curvature. These values were excluded. We found no penetration (0 mm) in three cases: two of them with the pin-cone angles 50°/36°, both with the 0° two-pin rocker and prone approach, and the 40° two-pin rocker and prone approach, respectively; and one with the pin-cone angles 50°/25° with the 0° two-pin-rocker and pterional approach. Apart from those cases, overall pin penetration depth ranged from 0.17 mm to 6.17 mm (mean: 1.23 mm, SD: 1.09 mm). No significant difference (*p* = 0.34) was identified correlating penetration depth to the angle of the different two-pin rockers. We recognized significant differences (*p* < 0.05) when the penetration depth was assigned to the craniotomy approach. The pterional approach caused an overall significantly higher penetration depth (mean: 1.68 mm, SD: 1.17 mm, pterional—middle fossa *p* = 0.019, pterional—prone *p* = 0.017) than the middle fossa (mean: 1.14 mm, SD: 1.25 mm, middle fossa—prone *p* = 0.78) and the prone (mean: 0.88 mm, SD: 0.59 mm) approach. The penetration depth of each test setup is illustrated in Fig. 6 for the single-pin side.

On the single-pin side only we observed factures in the neurocranium in four cases. Two of four fractures were caused by the pin configuration 36°/36° with 40° two-pin rocker, both middle fossa approach. One facture was caused by the pin configuration 36°/36° and one facture was caused by the pin configuration 55°/25°, both with the 0° two-pin rocker, both pterional approaches respectively. None of the fractures were noticed by the operator during the skull clamping procedure.

**Figure 7:**
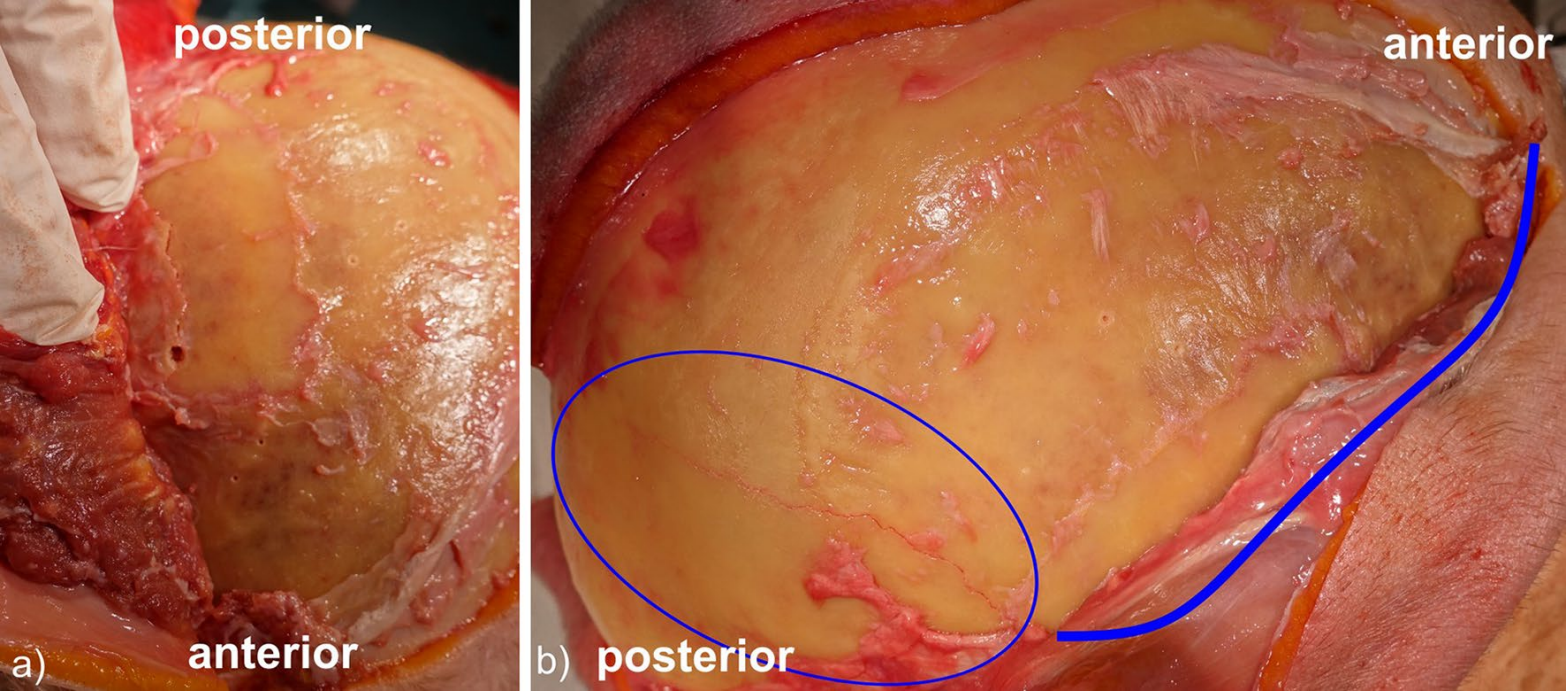
Optically recorded facture caused by application of pin force in a pterional approach using the pin configuration 36°/36° and the 0° two-pin rocker. (**a**) The start of fracture beginning form the penetration. (**b**) The path of fracture. The fracture is highlighted by a blue line and an ellipsoid.

While three factures reached a diameter of maximum 3.5 mm, one pterional pinning produced an extensive, dorsally projecting facture, originating from the pin-entry near the lateral orbital pillar at the junction of sphenoid and frontal bone, then traversing through the entire temporal bone and a large portion of the parietal bone, ultimately protracting towards the inion. The pin configuration 36°/36° and the 0° two-pin rocker were used. The fracture occurred during the last application of pin force to the respective cadaveric specimen. The fracture is depicted in Fig. 7.

### Penetration depth on two-pin side

**Figure 8:**
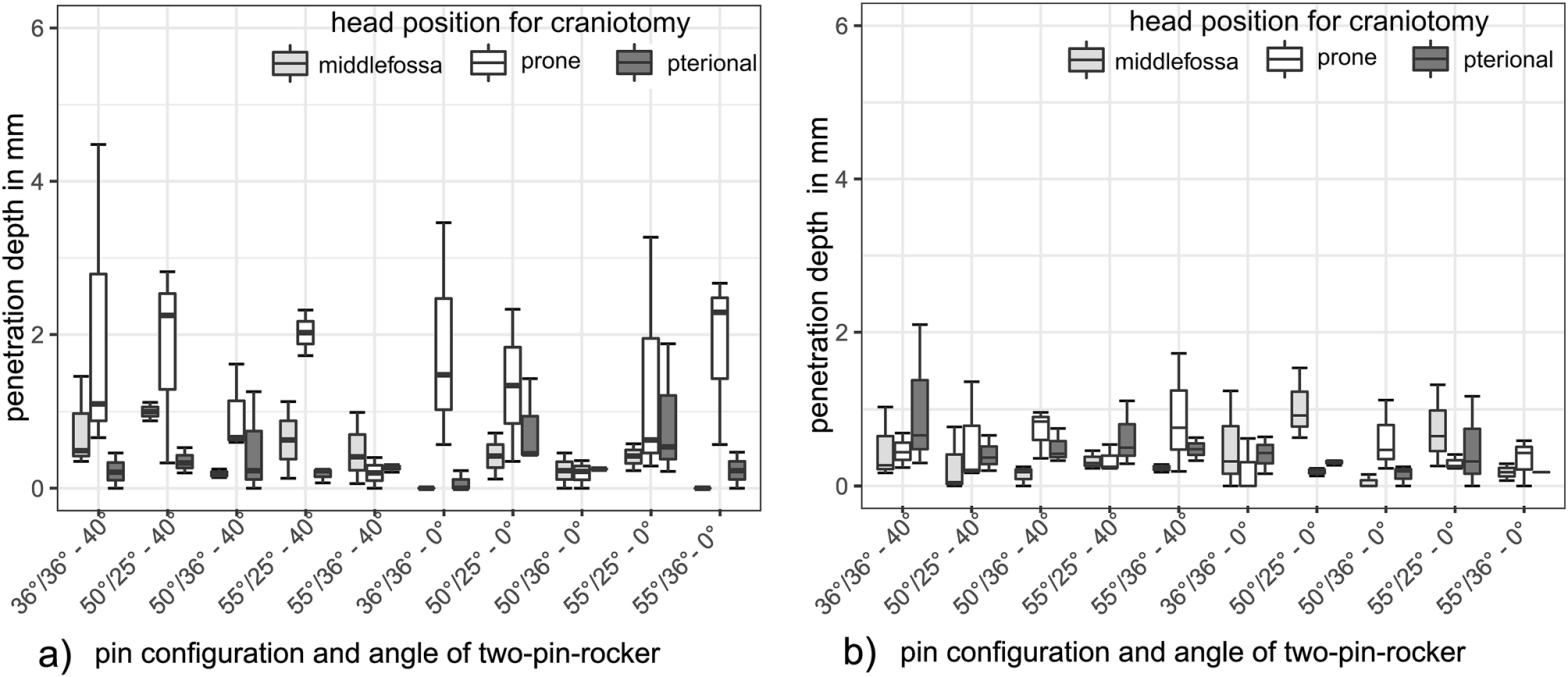
Penetration depth on two-pin side (1) and (2) for each test setup. The first number on the bottom describes the pin-cone angle on single-pin side in degree. The second number describes the pin-cone angle on two-pin side in degree. The third number represents the 40° or 0° two-pin rocker. (a) The results of two-pin side (1), The pins on the right respectively anterior. (**b**) The results of two-pin side (2), The pins on the left respectively posterior.

On the two-pin side, bone penetration depths could not be measured in ten cases for pin (1) and in six cases for pin (2) based on tangential impact of the pin. We registered no penetration (0 mm) in eleven cases on two-pin side (1) and in nine cases on two-pin side (2). Generally, pin (1) produced deeper bone penetrations (range 0.06–4.48 mm, mean: 0.83 mm, SD: 0.97 mm) than pin (2) (range 0.05–2.10 mm, mean: 0.47 mm, SD: 0.45 mm), please see Fig. 8.

The angle of the two-pin rocker (40° vs. 0°) had no influence on penetration depth neither on pin (1) nor on pin (2), (*p* = 0.44), respectively. The different head positions, however, produced significant penetration depth differences for pin (1) on the two-pin side only. More specifically, the prone position (mean: 1.51 mm, SD: 1.25 mm) resulted in a significantly (*p* = 0.001) deeper penetration of pin (1) than the middle fossa (mean: 0.54 mm, SD: 0.53 mm) and the pterional position (mean: 0.46 mm, SD: 0.56 mm). In the prone position there was also a significant difference (*p* = 0.001) in penetration depth between the more anteriorly located pin (1) (mean: 1.51 mm, SD: 1.25 mm) and pin (2) (mean: 0.46 mm, SD: 0.40 mm). Penetration depths of the two-pin side with respect to all variable set-ups are depicted in Fig. 8.

### Comparison of opposite pin-sides

**Figure 9:**
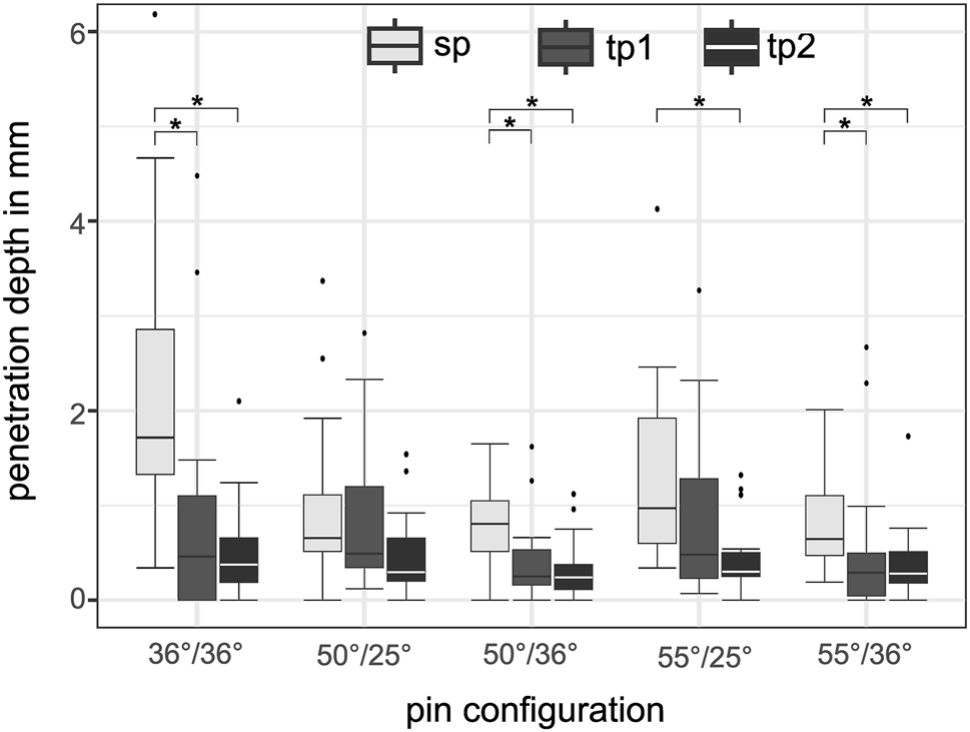
Penetration depth pin configuration. Boxplots marked with “sp” represents the penetration depth of all test setups of single-pin side. Boxplots marked with “tp1” or “tp2” represents the penetration depth of all test setups of two-pin side (1) or two-pin side (2). Significant differences of the penetration depth within a pin configuration are marked with*.

We compared the penetration depth of the single-pin side with two-pin sides (1) and (2) of each setup independent of the two-pin rocker angle. The differences were not significant (*p* > 0.05) with all three craniotomy approaches only using the pin configuration 50°/25°: middle fossa *p* = 0.38, pterional *p* = 0.11, prone *p* = 0.42 for pin (1) and *p* = 0.27 for pin (2). The *p*-values were separated for prone due to the significant difference as described in Penetration depth on two-pin side. The comparison of the single-pin side with two-pin sides (1) and (2) is demonstrated by Fig. 9.

## Discussion

The present study investigates skull penetration depths of skull pins with different pin-cone angles in a three-point HFD. In addition, it evaluates the effects of the head position at pinning, as well as the angulation of the rocker at the two-pin side of the HFD.

The results indicate that the currently used pin configuration, employing three uniform pins of 36°, results in an asymmetric bone-penetration distribution to the skull, thus potentially favoring slippage complications and bone fractures. The authors identify a non-uniform pin configuration that resulted in a quasi-homogenous skull penetration depth for all three pins. Notably, different head pinning positions entailed significantly varying skull penetration depths.

### Pin configuration for quasi-homogeneous penetration depth

The pin configuration 50°/25° resulted in quasi-homogeneous penetration depths with all head positions employed, while all other pin configurations tested (36°/36°; 50°/36°; 55°/25°; 55°/36°) produced significantly differing penetration depths.

It is assumed that bone penetration depth is a function of local pressure applied, given the two variables (1) outer force applied and (2) resistance/viscosity of the penetrated material remain stable. Consequently, considerably different bone penetration depths are indicative of an uneven pressure distribution, which is potentially undermining the stability of the system. The authors postulate that quasi-homogenous pin penetration depths represent a stability criterion for cranial pinning, rendering the head more resistant to external shear forces or torque.

### Influence of head position

The influence of the head position on pinning depth may be allocated to two main varying properties of the skull: (1) the local composition and thickness of the skull bone; (2) the local convexity of the skull bone.

1. It must be assumed that bone penetration depths are influenced by the local resistance and thickness of the skull, particularly the outer pars compacta. It is well known that the thickness of the skull varies regionally, with generally thicker layers in the occipital and parietal region and thinner layers in the temporal and frontobasal region consistent to recommended pinning-zone^8,12^. In addition, the thickness of the scalp and the presence of additional muscular layers differs regionally^12^. The surmountable soft tissue impediment may have contributary influences on pressure development.
2. Predetermined by the non-homogenous convexity of the skull there are parts of quasi-planar (lateral/temporal region) and parts of sharp convexity (particularly the mid-parieto-occipital region). The local bone curvature has influence on the impact angle of the pin, especially on the two-pin side, where angulation variance is restricted by the rigid angle of the two-pin rocker.

### Effects on single-pin side

Overall, we observed the deepest penetrations on the single-pin side with the pterional position. Here, the single-pin is mounted to the basal fronto-lateral bone, close to the lateral orbital pillar, where bone thickness is low. The pin may also impinge the anterior temporal bone lateral to the orbital pillar, as shown in the fracture example in Fig. 7, where bone thickness is even lower. Interestingly, for the 36°/36° pin configuration plus the 40° rocker isolated, we found the deepest penetrations on the single-pin side for the middle fossa approach. This finding is congruous with the results of Visentin et al.^9^. With the middle fossa approach, the single-pin hits the frontal bone basally near the air-filled sinuses, where bone density is also rather low. Most fractures occurred with the 36°/36° pin configuration. It may be hypothesized that this very pin configuration may lead to disproportionally high pressures on the single-pin side.

Based on the principle that force equals counterforce in a setting where the linearly opposing counterforce to the single-pin side is distributed between two-pins, local pressure to the single-pin side prevails, especially, when employing three uniform pin-cone angle.

### Effects on two-pin side

On the two-pin side we tested two two-pin rockers of different angulation (40° vs 0°). The two-pin rocker angle conditions the angle of impact to the bone on the two-pin side. The more orthogonally the axial force transmission from single-pin side to two-pin side, the better the anchoring of pinned head. Based on this consideration, we hypothesized that the 0° rocker may result in more favorable bone-penetration angles, particularly when pinning rather straight skull regions as is the case in the prone position. However, leastwise concerning penetration depths we did not register statistical differences between the two two-pin rockers. Mentionable, in the prone position the more anteriorly located pin (1) produced significantly deeper penetrations that the posterior pin (2). In the prone position the anterior pin impacts the anterior temporal region, where the corticalis is thinnest, while the posterior pin impacts the more resistant parietal bone.

### Limitations

We identified three limitations to this study. Even though we used fresh cadaveric specimens untreated by formaldehyde or other conservatives, it cannot be excluded that the mechanical properties of the scalp and skull bone may have changed within the postmortem period. Also, the cadaver cohort may not be representative for the average neurosurgical patient population. Although we were uninformed about the exact ages of the deceased, the cadaveric heads were of elderly people. It is well known that skull bone thickness increases with age^12,13^. Secondly, due to ethical reasons we used a limited sample size of eight cadaveric specimen. Those were pinned on rotation to repeat each test set-up three times. Additionally, the non-parametric Scheirer- Ray-Hare test was used, which has a lower power than other parametric analysis tests. Both factors may increase the statistical type two error. This implies that some statistically relevant differences may have been overlooked in this study. A third limitation was caused by the design geometry of the dial indicator. Its base provided only for orthogonal measurements of penetration depths. The penetration depth near sharp curvatures of the skull was not recorded.

## Conclusion

A novel pin configuration was obtained for a quasi-homogenous skull penetration using three-point HFDs. It is hypothesized that this pin configuration may increase the stability of the skull, reducing slippage complications and skull fractures. Further studies will follow to test the identified novel pin configuration against external shear forces and torque may be needed to confirm this as a stability criterion. The risk of incorrect placement of a pin with a pin-cone angle of 25° to the single-pin side (and reverse) and possible skin injuries by a not optimal pin diameter must be evaluated and if applicable reduced by technical solutions.

## Data Availability

The datasets generated or analyzed during the current study are available in the figshare repository, https://doi.org/10.6084/m9.figshare.23267672.
